# Advisory Committee on Immunization Practices Recommended Immunization Schedule for Children and Adolescents Aged 18 Years or Younger — United States, 2021

**DOI:** 10.15585/mmwr.mm7006a1

**Published:** 2021-02-12

**Authors:** A. Patricia Wodi, Kevin Ault, Paul Hunter, Veronica McNally, Peter G. Szilagyi, Henry Bernstein

**Affiliations:** ^1^Immunization Services Division, National Center for Immunization and Respiratory Diseases, CDC; ^2^University of Kansas Medical Center, Kansas City, Kansas; ^3^Department of Family Medicine and Community Health, University of Wisconsin, Madison, Wisconsin; ^4^Fanny Strong Foundation, West Bloomfield, Michigan; ^5^Department of Pediatrics, University of California Los Angeles, Los Angeles, California; ^6^Zucker School of Medicine at Hofstra/Northwell, Hempstead, New York; ^7^Department of Pediatrics, Cohen Children’s Medical Center, New Hyde Park, New York.

At its October 2020 meeting, the Advisory Committee on Immunization Practices* (ACIP) approved the 2021 Recommended Child and Adolescent Immunization Schedule for Ages 18 Years or Younger. After Emergency Use Authorization of Pfizer-BioNTech COVID-19 vaccine by the Food and Drug Administration (FDA), ACIP issued an interim recommendation for use of Pfizer-BioNTech COVID-19 vaccine in persons aged ≥16 years at its December 12, 2020, meeting (*1*). In addition, ACIP approved an amendment to include COVID-19 vaccine recommendations in the child and adolescent immunization schedule. After Emergency Use Authorization of Moderna COVID-19 vaccine by FDA, ACIP issued an interim recommendation for use of Moderna COVID-19 vaccine in persons aged ≥18 years at its December 19, 2020, emergency meeting (*2*).

The 2021 child and adolescent immunization schedule summarizes ACIP recommendations, including several changes from the 2020 immunization schedule^†^ on the cover page, two tables, and notes found on the CDC immunization schedule website (https://www.cdc.gov/vaccines/schedules). Health care providers are advised to use the tables and the notes together. This immunization schedule is recommended by ACIP (https://www.cdc.gov/vaccines/acip) and approved by CDC (https://www.cdc.gov), the American Academy of Pediatrics (https://www.aap.org), the American Academy of Family Physicians (https://www.aafp.org), the American College of Obstetricians and Gynecologists (https://www.acog.org), the American College of Nurse-Midwives (https://www.midwife.org), the American Academy of Physician Assistants (https://www.aapa.org), and the National Association of Pediatric Nurse Practitioners (https://www.napnap.org).

ACIP’s recommendations on use of each vaccine are developed after in-depth reviews of vaccine-related data, including the epidemiology and societal impacts, vaccine efficacy and effectiveness, vaccine safety, quality of evidence, feasibility of program implementation, and economic analyses of immunization policy (*3*). The child and adolescent immunization schedule is published annually to consolidate and summarize updates to ACIP recommendations on vaccination of children and adolescents, and to assist health care providers in implementing current ACIP recommendations. The use of vaccine trade names in this report and in the child and adolescent immunization schedule is for identification purposes only and does not imply specific product endorsement by ACIP or CDC.

For further guidance on the use of each vaccine, including contraindications and precautions, and any updates that might occur between annual updates to the child and adolescent immunization schedule, health care providers are referred to the respective ACIP vaccine recommendations at https://www.cdc.gov/vaccines/hcp/acip-recs.^§^ Printable versions of the 2021 child and adolescent immunization schedule and ordering instructions are available at https://www.cdc.gov/vaccines/schedules/hcp/imz/child-adolescent.html.

## Changes in the 2021 Child and Adolescent Immunization Schedule

Vaccine-specific changes in the 2021 child and adolescent immunization schedule for children and adolescents aged 18 years or younger include new or updated ACIP recommendations for influenza vaccine (*4*) meningococcal serogroups A, C, W, and Y (MenACWY) vaccines (*5*), and COVID-19 vaccines (*1**,**2*). Changes also include clarification of the recommendations for diphtheria and tetanus toxoids and acellular pertussis vaccine (DTaP), *Haemophilus influenzae* type b vaccine (Hib), hepatitis A vaccine (HepA), hepatitis B vaccine (HepB), human papillomavirus vaccine (HPV), pneumococcal vaccines (PCV13 and PPSV23), measles, mumps, and rubella virus vaccine (MMR), tetanus toxoid, reduced diphtheria toxoid, and acellular pertussis vaccine (Tdap), and varicella vaccine (VAR). Following are the changes to the cover page, Tables 1 and 3, and the Vaccine Notes.

### Cover page

- The American Academy of Physician Assistants and the National Association of Pediatric Nurse Practitioners have been added to the list of organizations that approve the child and adolescent immunization schedule.
- MenACWY-TT (MenQuadfi) and Diphtheria and Tetanus Toxoids and Acellular Pertussis Vaccine Adsorbed, Inactivated Poliovirus, *Haemophilus* b Conjugate and Hepatitis B Vaccine (Vaxelis) have been added to the table of vaccine abbreviations/trade names.
- The abbreviation for live attenuated influenza vaccine (LAIV) was changed to LAIV4.

### Table 1

- **HepB row**: Arrows have been added to clarify the recommended ages for administering the second dose.
- **LAIV:** The abbreviation was changed to LAIV4.

### Table 3

- **Legend: **The text that defines the red box has been edited to include “Vaccinate after pregnancy.” The text now reads “Not recommended/contraindicated—vaccine should not be administered. *Vaccinate after pregnancy.”
- **LAIV:** The abbreviation was changed to LAIV4.
- **MMR row:** An asterisk has been added in the pregnancy column. The asterisk links to the descriptive text “*Vaccinate after pregnancy” in the red box of the table’s legend.
- **VAR row:** An asterisk has been added in the pregnancy column. The asterisk links to the descriptive text “*Vaccinate after pregnancy” in the red box of the table’s legend.
- **HPV row: **The color for the pregnancy column has been changed from pink to red; an asterisk has also been added. The asterisk links to the descriptive text “*Vaccinate after pregnancy” in the red box of the table’s legend.

### Notes

• **Additional Information:** The section has been updated to include COVID-19 vaccination recommendations.

• **DTaP: **A “Special situations” section has been added that contains information regarding the recommendation for use of DTaP vaccine in wound management.

• **Hib: **Text has been added to clarify the recommendations for catch-up vaccination. A bullet has been added to indicate that no further doses are needed if a dose was administered at age ≥15 months.

• **HepA:** The note was updated to clarify information on the accelerated 4-dose series of combined HepA-HepB vaccine. The fourth dose at month 12 is a booster dose.

• **HepB: **Additional text has been added to emphasize the birth dose in the vaccination note. The sentence on recommendations for infants born to an HBsAg-negative mother and weighing <2,000 g has been updated with language to provide further clarification regarding when the vaccine can be administered.

• **HepB:** The note was updated to clarify information on the accelerated 4-dose series of combined HepA-HepB vaccine. The fourth dose at month 12 is a booster dose.

• **HPV:** The note has been updated to clarify that if the vaccination schedule is interrupted, the series does not need to be restarted.

• **Influenza vaccination: **The note has been updated to reflect the recommendations for the 2020–21 influenza season. The “Special situations” section was updated with language for persons who have egg allergy with symptoms other than hives, and two new bullets were added with information on severe allergic reactions after influenza vaccination. The abbreviation LAIV was changed to LAIV4. In addition, the bullets that outline circumstances under which LAIV4 should not be used were updated to include children aged <2 years, and more detailed information on the use of LAIV4 after receipt of influenza antiviral medications to account for newer antivirals with longer half-lives was added.

• **MenACWY:** MenACWY-TT (MenQuadfi) has been added to the list of vaccines in the sections on routine vaccination, catch-up vaccination, and special situations. In addition, the “Special situations” section has been updated with information on the recommendations for the use of MenACWY-CRM (Menveo) in infants who received dose 1 at age 3–6 months.

• **Pneumococcal vaccination: **Text has been added to the “Special situations” section of the note to clarify the recommendations for administering PPSV23 after PCV13.

• **Tdap: **A “Special situations” section has been added to the note that contains information regarding the recommendation for use of Tdap vaccine in wound management.

## Additional Information

The Recommended Child and Adolescent Immunization Schedule, United States, 2021 is available at https://www.cdc.gov/vaccines/schedules/hcp/imz/child-adolescent.html. The full ACIP recommendations for each vaccine are also available at https://www.cdc.gov/vaccines/hcp/acip-recs. All vaccines identified in Tables 1, 2, and 3 (except DTaP, rotavirus, and poliovirus vaccines) also appear in the Recommended Adult Immunization Schedule for Ages 19 Years or Older, United States, 2021, available at https://www.cdc.gov/vaccines/schedules/hcp/imz/adult.html. The notes for vaccines that appear in both the child and adolescent immunization schedule and the adult immunization schedule have been harmonized to the greatest extent possible.

## Footnotes

* Recommendations for routine use of vaccines in children and adolescents are developed by ACIP, a federal advisory committee chartered to provide expert external advice and guidance to the CDC director on use of vaccines and related agents for the control of vaccine-preventable diseases in the civilian population of the United States. Recommendations for routine use of vaccines in children and adolescents are harmonized to the greatest extent possible with recommendations made by the American Academy of Pediatrics (AAP), the American Academy of Family Physicians (AAFP), the American College of Obstetricians and Gynecologists (ACOG), the American College of Nurse-Midwives (ACNM), the American Academy of Physician Assistants (AAPA), and the National Association of Pediatric Nurse Practitioners (NAPNAP). ACIP recommendations approved by the CDC director become agency guidelines on the date published in the *Morbidity and Mortality Weekly Report* (MMWR). Additional information about ACIP is available at https://www.cdc.gov/vaccines/acip

† Past immunization schedules are available at https://www.cdc.gov/vaccines/schedules/past.html

§ CDC encourages organizations to use syndication as a more reliable method for displaying the most current and accurate immunization schedules on an organization's website rather than copying these schedules to their websites. Use of content syndication requires a one-time step that ensures an organization’s website displays current schedules as soon as they are published or revised; instructions for the syndication code are available on CDC’s website (https://www.cdc.gov/vaccines/schedules/syndicate.html). CDC also offers technical assistance for implementing this form of content syndication (e-mail request to ncirdwebteam@cdc.gov).
